# Abandonment of intravenous volume expansion after preoperative receipt of α-blockers in patients with adrenal pheochromocytoma was not an independent risk factor for intraoperative hemodynamic instability

**DOI:** 10.3389/fendo.2023.1131564

**Published:** 2023-04-21

**Authors:** Kun-wu Yan, Xiao-fei Tian, Yan-ni Wu, Meng Cai, Ming-tao Guo

**Affiliations:** ^1^ Department of Urology, Handan First Hospital, Handan, China; ^2^ Department of General Surgery, Handan First Hospital, Handan, China; ^3^ Department of Pharmaceutical Technology, Faculty of Pharmacy, Heze Domestic Professional College, Heze, China; ^4^ Department of Oncology, Handan First Hospital, Handan, China

**Keywords:** intravenous volume, expansion, α-blockers, adrenal pheochromocytoma, hemodynamic

## Abstract

**Background:**

There is no consensus on whether intravenous rehydration must be added after preoperative phenoxybenzamine (PXB) administration for pheochromocytoma. The aim of this study is to investigate whether abandonment of intravenous volume expansion after PXB administration is associated with intraoperative hemodynamic instability.

**Methods:**

83 Patients with pheochromocytoma received surgical treatment in the Department of Urology, Handan First Hospital, between October 2014 and July 2022. All patients were subclassified into either the hemodynamic stability group (HS group) or the hemodynamic instability group (HU group) according to whether intraoperative hemodynamic instability occurred, with 51 cases in HS group and 32 cases in HU group. Differences in data between the two groups were examined, and the risk factors for intraoperative hemodynamic instability were analyzed using logistic regression.

**Results:**

The results of the analysis showed no statistically significant differences in age, sex, location of the tumor, surgical method, body mass index (BMI) ≥ 24 kg/m^2^, blood and urine catecholamine test results, preoperative oral PXB followed by combined intravenous volume expansion, proportion of patients with hypertension or diabetes mellitus or coronary heart disease between the two groups (P>0.05). The size of the tumor in the HS group was smaller than that in the HU group (5.3 ± 1.9 cm vs 6.2 ± 2.4 cm P=0.010). Multivariate analyses demonstrated that abandonment of intravenous volume expansion after preoperative receipt of α-blockers in patients with adrenal pheochromocytoma was not an independent risk factor for intraoperative hemodynamic instability. Only the tumor size (P=0.025) was an independent risk factor for intraoperative hemodynamic instability.

**Conclusion:**

The purpose of general preoperative intravenous fluid expansion is to prevent hypotension after the tumor has been resected. In the current study, we indicated that preoperative management of pheochromocytomas using the α-blocker PXB in combination with intravenous volume expansion does not further reduce the risk of intraoperative hemodynamic instability or postoperative complications compared with oral PXB alone. Therefore, our study supports preoperative management of pheochromocytoma with a single α-blocker, PXB, as sufficient.

## Introduction

Pheochromocytoma is a rare catecholamine-producing neuroendocrine tumor that originates from chromophores in the adrenal medulla, and can cause a variety of clinical symptoms (1). In mild cases, the symptoms may be silent, localized, or nonspecific; in typical cases, the symptoms may be paroxysmal hypertension with headache, palpitations, and excessive sweating, while in severe cases, they may cause hemodynamic instability and end-organ damage or insufficiency (2). The release of catecholamines from pheochromocytoma can cause a range of clinical manifestations, including hypertension, sweating, and tachycardia (3). Currently, surgical removal of the tumor is the only available radical approach for its treatment; unfortunately, intraoperative management of the tumor can lead to uncontrolled release of catecholamine, which may result in unstable intraoperative hemodynamic changes, including fatal hypertensive crisis and arrhythmias (4). Therefore, patients with a suspected or confirmed diagnosis of pheochromocytoma should undergo adequate preoperative preparation to improve the safety of the procedure. Bihain et al. found that, after reviewing nearly 50 years of literature on the use of antihypertensive drugs in patients with pheochromocytoma, the proportion of patients suffering with perioperative complications decreased from 69% to 3% (5). Buitenwerf et al. used α-blockers for preoperative management of 134 patients with pheochromocytoma (6). The 30-day cardiovascular complication rate was 8.8% and 6.9% and there was no mortality after 30 days. The consensus recommendation is that all patients with pheochromocytoma should receive appropriate preoperative medical management to block the effects of circulating catecholamines.

Currently, the main preoperative preparatory drugs for pheochromocytoma include α-blockers for blood pressure control and beta-blockers for heart rate stabilization. Although α-blockers were first administered to patients in the middle of the last century, they are administered before pheochromocytoma resection (7) and have been recommended for reducing the risk of intraoperative hypertension since their introduction. Blockade with α-blockers is currently the first choice for preoperative blockade in patients with functional pheochromocytoma (1). α-blockers are divided into two categories: selective and non-selective. Zawadzka et al. reported that non-selective α-blockade was more effective in preventing intraoperative blood pressure fluctuations while maintaining comparable risk of both intraoperative and postoperative hypotension (8). The American College of Endocrinology Clinical Practice Guidelines recommend that all pheochromocytoma patients receive α-adrenergic receptor blockers for 7-14 days preoperatively to reduce surgical mortality and postoperative complication rates. The results of another study provided evidence of more complete preoperative preparation, patients should consume fluids and be instructed to eat foods high in sodium and to increase blood volume as soon as they are on α-blockers (9). Continuous use of phenoxybenzamine along with intravenous saline until 1 day before surgery has also been reported (6, 10). At present, the effect of oral α-blockers combined with intravenous volume expansion on perioperative hemodynamics is inconclusive, and it is worth exploring whether intravenous fluid expansion is necessary after preoperative oral phenoxybenzamine. Therefore, in our medical center, we use phenoxybenzamine (PXB) as a preoperative preparation drug for pheochromocytoma patients; therefore, the aim of this study is to investigate whether perioperative hemodynamics are associated with preoperative oral phenoxybenzamine followed by intravenous volume expansion.

## Patients and methods

This study was a retrospective analysis and included patients with pheochromocytoma who were hospitalized at Handan First Hospital from October 2014 to July 2022. A total of 89 patients were treated surgically, of whom 6 were excluded, including 4 patients with incomplete data, 1 patient with tumor recurrence, and 1 patient with an extra-adrenal tumor. A total of 83 patients were finally screened. pheochromocytoma was considered by qualitative diagnosis (24-hour urinary catecholamines, plasma catecholamines, 24-hour urinary fractionated metanephrines, plasma metanephrines) and local diagnosis (CT plain + enhanced, MRI), and was supported by postoperative pathology. patients with a postoperative pathological diagnosis not supporting pheochromocytoma. The ethical review committee approved the study at Handan First Hospital, and the requirement for informed consent was waived due to the retrospective design.

Before 2018, patients undergoing pheochromocytoma resection were treated with the α-blocker PXB 14 days prior to surgery and intravenous volume expansion 3 days prior to surgery. Since 2018, we are gradually using α-blocker PXB alone and discontinuing preoperative intravenous volume expansion in patients undergoing pheochromocytoma resection. 41 of the 83 patients did not receive intravenous volume expansion. Before surgery, all patients completed at least one catecholamine test, such as for norepinephrine, epinephrine, and dopamine. Since 2017, we have performed blood and urine metanephrines tests in patients with suspected pheochromocytoma. Therefore, 37 of the 83 patients did not receive metanephrines testing. The patients were considered to have tested positive for catecholamines if one of their tests for norepinephrine, epinephrine and dopamine was above the normal range, If all of them were within the normal range, the test is considered negative. Of the 83 cases we collected, 54 patients tested positive for catecholamines (65.1%) and 29 patients tested negative for catecholamines (34.9%).

### Preoperative medications

All patients received non-selective α-blockade PXB as part of preoperative medical preparation for at least 2 weeks. The initial dose of PXB was 10 mg orally twice daily, and the maximum dose was 10 mg orally five times daily. Patients were hospitalized in our endocrinology department or followed up regularly in outpatient clinics to regulate their blood pressure. Clinicians increased or decreased the dose of PXB intake depending on the patient’s daily blood pressure fluctuations. Hypertension defined as systolic blood pressure (SBP) > 140 mmHg or diastolic blood pressure (DBP) > 90 mmHg. In accordance with the 2022 Endocrine Society recommendations, the preoperative patient’s target blood pressure was controlled to less than 130/80 mmHg and the target heart rate to approximately 60-70 beats per minute when sitting (1). and changes in peripheral circulation, such as warmth in the extremities, were observed. Other drugs for hypertension, such as β-blockers (BB), calcium channel blockers (CCB), angiotensin-converting enzyme inhibitors (ACEI), and angiotensin receptor blockers (ARB), were considered when needed. All patients were admitted to the urology department 3 days before surgery. Patients who needed combined intravenous volume expansion were given 1000-2000 ml of saline intravenously 1 day before the procedure.

### Surgical techniques

**Figure 1:**
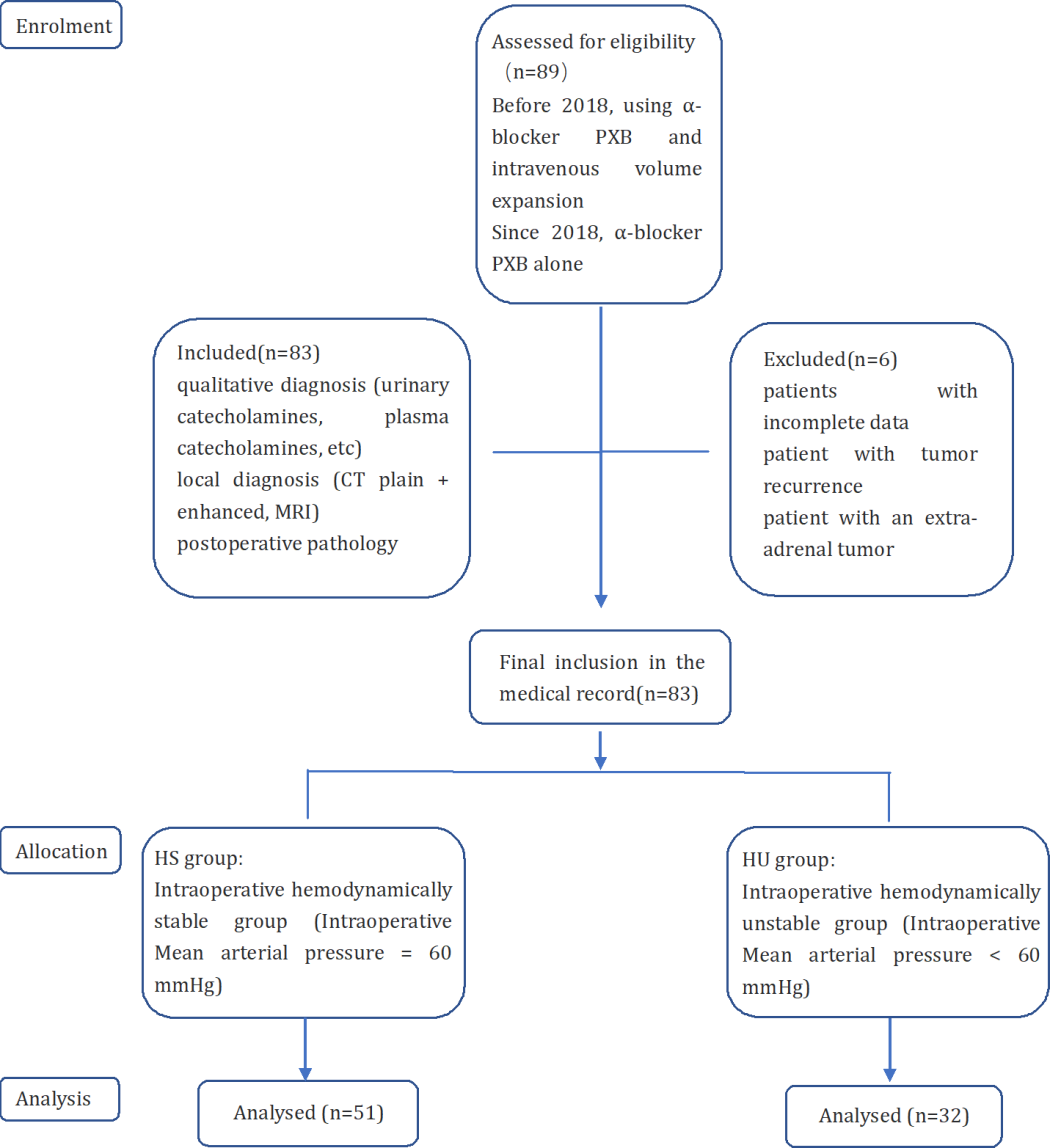
Flow chart of the study grouped according to hemodynamic stability or not.

All patients underwent adrenal tumor resection under general anesthesia. The surgical approach (open versus laparoscopic) was determined based on tumor size, relationship to adjacent organs, and history of previous abdominal surgery. The anesthesiologist routinely inserted an arterial catheter into the patient’s radial artery after anesthesia induction to monitor arterial pressure. The computer automatically recorded blood pressure measurements every 10 seconds. Haemodynamic variables, including BP and HR, were collected from the electronic anaesthetic chart, the intraoperative hemodynamic instabilities were defined as an intraoperative mean arterial pressure <60 mmHg (11). The main observation was whether there was intraoperative hemodynamic stability, followed by complications directly related to hemodynamic stability, including new-onset arrhythmias, postoperative hypotension, and need for antihypertensive drugs (12). Patients were divided into the hemodynamically stable group (HS group) and the hemodynamically unstable group (HU group) according to whether they were hemodynamically stable intraoperatively (**Figure 1**).

### Statistical analysis

IBM SPSS statistics v26.0. Software was used to analyze the data. Continuous variables were presented as means and standard deviations. Group comparisons for continuous variables were performed by the t-test for approximately normally distributed data or the Mann‐Whitney U-test for non‐normally distributed data. Category variables were presented as numbers and percentages. Group comparisons for categorical variables were performed by the χ^2^-test. Binary logistic regression analysis was used to determine predictive risk factors of hemodynamic instability. P < 0.05 (two‐tailed tests) was defined as a statistically significant difference.

## Results

This study included 30 men and 53 women with a mean age of 45 at the time of surgery. The patient characteristics in the two groups were comparable as shown in **Table 1**. The tumor size in the HS group [(5.3 ± 1.9) cm] was smaller than that in the HU group [(6.2 ± 2.4) cm], and the difference between the two groups was statistically significant (P=0.010). There were no significant differences in age, tumor size, tumor location, surgical method, preoperative oral PXB followed by combined intravenous volume expansion, BMI ≥ 24 kg/m^2^, hypertension history, mellitus history, coronary artery disease history, blood catecholamines and urine catecholamines in the two groups.

**Table 1:** Clinical characteristics of patients with adrenal pheochromocytoma.

	Overall (n = 83)	HS group (n = 51)	HU group (n = 32)	P value
Age, years (mean ± SD)	45.2 ± 8.5	44.5 ± 8.1	46.7 ± 9.2	0.546
Sex, n (%)				0.790
Male	30 (36.1)	19 (37.3)	11 (34.4)	
Female	53 (63.9)	32 (62.7)	21 (65.6)	
Size of tumor, cm (mean ± SD)	5.6 ± 2.2	5.3 ± 1.9	6.2 ± 2.4	0.010
Location of tumor, n (%)				0.710
Left	24 (28.9)	14 (27.5)	10 (31.2)	
Right	59 (71.1)	37 (72.5)	22 (68.8)	
Surgery method, n (%)				0.625
Open	31 (37.3)	18 (35.3)	13 (40.6)	
Laparoscopy	52 (62.7)	33 (64.7)	19 (59.4)	
Catecholamine test, n (%)				0.390
Positive	54 (65.1)	35 (68.6)	19 (59.4)	
negative	29 (34.9)	16 (31.4)	13 (40.6)	
Preoperative oral PXB followed by combined intravenous volume expansion, n (%)	42 (50.6)	27 (52.9)	15 (46.9)	0.591
BMI ≥ 24 kg/m^2^, n (%)	49 (59.0)	31 (60.8)	18 (56.2)	0.683
Comorbidities				
Previous hypertension history, n (%)	67 (81.0)	42 (82.3)	25 (78.1)	0.635
Diabetes mellitus history, n (%)	14 (16.9)	8 (16.7)	6 (18.8)	0.717
Coronary artery disease, n (%)	7 (8.4)	5 (9.8)	2 (6.2)	0.571
Urine norepinephrine, nmol/24h	298 ± 59	281 ± 53	323 ± 67	0.620
Urine epinephrine, nmol/24h	34 ± 15	31 ± 12	38 ± 18	0.172
Urine dopamine, nmol/24h	2138 ± 348	2076 ± 299	2232 ± 392	0.283
Blood norepinephrine, pmol/L	2672 ± 487	2458 ± 437	2814 ± 543	0.664
Blood epinephrine, pmol/L	95 ± 19	94 ± 17	97 ± 22	0.322
Blood dopamine, pmol/L	67 ± 27	62 ± 24	74 ± 30	0.401

Data are presented as the mean standard deviation, median (interquartile range), or number (percentages).

BMI, body mass index; PXB, phenoxybenzamine (mg/d).

We further compared the preoperative application of α-blockers and other medications in the two groups (**Table 2**). The differences in medicinal use between the two groups of patients were not statistically significant. Moreover, age, tumor size, tumor location, surgical method, preoperative oral PXB followed by combined intravenous volume expansion, BMI ≥ 24 kg/m^2^, hypertension history, mellitus history, coronary artery disease history, blood catecholamines and urine catecholamines were subjected to multifactorial logistic regression analysis. Only tumor size was an independent risk factor for intraoperative hemodynamic instability (**Table 3**).

**Table 2:** Preoperative medication for patients with adrenal pheochromocytoma.

	Overall (n = 83)	HS group (n = 51)	HU group (n = 32)	P Value
Average dose of PXB (mean ± SD)	32.6 ± 9.4	31.3 ± 9.1	33.7 ± 9.8	0.127
Patients taking β-blockers, n (%)	14 (16.9%)	9 (17.6%)	5 (15.6%)	0.685
Patients taking ACEIs^a^, n (%)	5 (6.0%)	4 (7.8%)	1 (3.1%)	0.685
Patients taking ARBs^b^, n (%)	1 (1.2%)	0 (0.0%)	1 (3.1%)	0.813
Patients taking CCBs, n (%)	21 (25.3%)	15 (29.4%)	6 (18.8%)	0.277

Data are presented as the mean standard deviation, median (interquartile range), or number (percentages).

PXB, phenoxybenzamine (mg/d); ACEIs, angiotensin-converting enzyme inhibitors; ARBs, angiotensin receptor blockers; CCBs, calcium channel blockers.

a Continuity Correction was used for analysis.

b Fisher’s Exact Test was used for analysis.

**Table 3:** Multifactorial logistic regression analysis of intraoperative hemodynamic instability in patients with adrenal pheochromocytoma.

	B	S.E.	Wald	P	OR	95% CI
Age	-0.141	0.158	0.789	0.374	0.869	0.637~1.185
Sex (1=male)	-0.451	1.341	0.113	0.737	0.637	0.046~8.829
Size of tumor	1.490	0.677	4.847	0.028	4.439	1.178~16.731
Location of tumor (1=left)	-0.778	1.596	0.238	0.626	0.459	0.020~10.491
Surgery method (1=open)	-0.268	1.286	0.044	0.835	0.765	0.061~9.511
Preoperative oral PXB alone	0.630	1.312	0.230	0.631	1.878	0.143~24.592
BMI ≥ 24 kg/m^2^	-0.051	0.599	0.007	0.932	0.950	0.294~3.075
Previous hypertension history	-0.505	0.671	0.566	0.452	0.604	0.162~2.248
Diabetes mellitus history	0.938	0.843	1.240	0.266	2.556	0.490~13.329
Coronary artery disease	-1.282	1.141	1.264	0.261	0.277	0.030~2.594
Urine norepinephrine	0.024	0.016	2.238	0.135	1.025	0.993~1.058
Urine epinephrine	0.144	0.104	1.899	0.168	1.154	0.941~1.416
Urine dopamine	0.003	0.003	1.339	0.247	1.003	0.998~1.008
Blood norepinephrine	0.005	0.003	3.119	0.077	1.005	0.999~1.012
Blood epinephrine	0.076	0.072	1.134	0.287	1.079	0.938~1.242
Blood dopamine	-0.094	0.063	2.193	0.139	0.911	0.804~1.031

BMI, body mass index; PXB, phenoxybenzamine (mg/d).

There were 2 postoperative complications in the HS group (both were postoperative fat liquefaction of the wound) and 2 postoperative complications in the HU group (1 postoperative subcutaneous hematoma and 1 poor wound healing due to infection), and all patients had no tumor recurrence or metastasis at the 3-month postoperative follow-up. The difference was not statistically significant (χ^2 = ^0.323, P=0.614).

## Discussion

The main risk for patients with pheochromocytoma is intraoperative hemodynamic instability. Different studies have adopted reference standards for determining intraoperative hemodynamic stability. A common reference standard is the presence of mean arterial pressure less than a certain value, such as a mean arterial pressure < 60 mmHg. By analyzing the electronic medical records of 114 patients with pheochromocytoma, Kim et al. found that one or more episodes of mean blood pressure < 60 mmHg or systolic blood pressure > 200 mmHg during surgery was a high-risk factor for postoperative cardiovascular events (11). The reason for this condition is the increase of systolic blood pressure due to the release of catecholamines from the tumor into the bloodstream caused by compression of the tumor during surgery, and the decrease of mean arterial pressure due to insufficient establishment of collateral circulation after tumor removal. Combining the literature and clinical experience (13, 14), In our study, after initial sample size estimation, we included a total of 83 patients, intraoperative mean arterial pressure <60 mmHg is considered to be hemodynamically unstable.

According to reports, Huang had collected clinical data of 136 patients with pheochromocytoma who underwent laparoscopic adrenalectomy, and found that tumor size and previous history of hypertension were associated with intraoperative hemodynamic instability by multivariate logistic regression analysis (15). Pisarska-Adamczyk retrospectively analyzed data from 96 patients undergoing laparoscopic adrenalectomy and found that adrenal tumor size and diabetes mellitus were associated with hemodynamic instability during pheochromocytoma resection (16). Ma evaluated the relationship between preoperative parameters and the incidence of intraoperative hemodynamic instability in 428 patients with pheochromocytoma and found that tumor size and high urinary epinephrine levels were tumor-related factors for intraoperative hemodynamic instability (17). History of hypertension and diabetes mellitus and urinary epinephrine levels were not found to be associated with intraoperative hemodynamic stability in patients in our study. Larger tumours have been shown to significantly release catecholamines during pheochromocytoma removal and as a result to increase the number of intraoperative hypertensive episodes. Also as tumor size increases, the potential for intraoperative blood loss increases (16, 18). It is therefore not surprising that larger tumors are more prone to intraoperative hemodynamic fluctuations.

The 2022 year of Personalized Management of Pheochromocytoma and Paraganglioma published by The Endocrine Society (TES) recommends preoperative α-blockers for patients with secretory pheochromocytoma to reduce the incidence of perioperative complications, including intraoperative hemodynamic crises (1). PXB is a nonselective α-blocker that has been widely used in the clinic to help minimize intraoperative hemodynamic instability and to help control intraoperative catecholamine fluctuations (19).

Previous studies on this issue did not include the important factor of whether intravenous rehydration was available before the operation (18). This is probably because, in the past, such patients were usually started on intravenous saline after a period of preoperative oral α-blockers (6, 9). Currently, the duration of preoperative intravenous rehydration is based on previous experience and varies by institution. The earliest intravenous saline administration as preoperative management at our center was 3 days prior to surgery. some studies have reported the duration of preoperative intravenous rehydration as 1 week or even 2 weeks (6, 10). Preoperative intravenous rehydration was widely used in many medical centers, however its usefulness has not been well evaluated. Hao found that in patients with pheochromocytoma, preoperative intravenous rehydration did not optimize perioperative hemodynamics or improve early outcomes (20). Preoperative intravenous rehydration failed to optimize perioperative hemodynamics or improve early outcome and this approach may increase the burden on the kidneys and heart. In this study, we collected data from 83 patients with pheochromocytoma and subclassified them into two groups according to intraoperative hemodynamics. Comparing the data between the two groups, we found no significant differences in intraoperative hemodynamic changes or incidence of postoperative hypotension between patients with pheochromocytoma receiving preoperative α-blockers alone and those receiving α-blockers combined with intravenous volume expansion. The binary logistic regression shows that it did not show any association between hemodynamic instability and preoperative intravenous rehydration.

Of course, there were some limitations in this study. First, this study was a retrospective analysis of data from a single center. Related selectivity and recall bias may have been influential, and further external validation (especially from non-Chinese teams) would be significant. Second, our study did not consider using other types of α-blockers in the perioperative period and the anesthesiologists did not follow a uniform protocol to control intraoperative hemodynamic instability. Finally, Some scholars have used the LASSO regression model for bioinformatics articles and achieved good effects (21, 22), which warrants the application of this model to better predict the risk factors for hemodynamic instability.

## Conclusions

In conclusion, during the preoperative preparation of patients with pheochromocytoma, oral α-blockers plus intravenous volume expansion did not further reduce the incidence of intraoperative hemodynamic instability compared with oral α-blockers alone. Therefore, patients with adrenal pheochromocytoma are advised to take α-blockers alone preoperatively, with the addition of other antihypertensive agents to help stabilize blood pressure and expand blood volume if necessary. Since this study is a single-center retrospective study, selection bias exists, and the conclusions need to be further validated in a large sample of prospective randomized controlled studies.

## Data availability statement

The raw data supporting the conclusions of this article will be made available by the authors, without undue reservation.

## Ethics statement

Ethical review and approval was not required for the study on human participants in accordance with the local legislation and institutional requirements. Written informed consent from the patients was not required to participate in this study in accordance with the national legislation and the institutional requirements.

## Author contributions

K-WY and X-FT: first author. Y-NW, MC, M-TG: Senior authorship. All authors contributed to the article and approved the submitted version.
